# *N*-Ethyl-2-[1-(2-hy­droxy­naphthalen-1-yl)ethyl­idene]hydrazinecarbo­thio­amide

**DOI:** 10.1107/S1600536814012057

**Published:** 2014-05-31

**Authors:** Brian J. Anderson, Zachary A. Shalit, Jerry P. Jasinski

**Affiliations:** a Department of Chemistry, Keene State College, 229 Main Street, Keene, NH 03435-2001, USA

## Abstract

In the title compound, C_15_H_17_N_3_OS, the dihedral angle between the mean planes of the 2-hy­droxy­napthyl ring system and the hydrazinecarbo­thio­amide group is 73.7 (3)°. In the crystal, weak O—H⋯S and C—H⋯O inter­actions and π–π stacking inter­actions involving one of the hy­droxy­napthyl rings with a centroid–centroid distance of 3.6648 (14) Å are observed, forming infinite chains along [010]. In addition, N—H⋯S inter­actions occur.

## Related literature   

For the biological activity of thio­semicarbazones, see: Chellan *et al.* (2010 ▶). For binding motifs of thio­semicarbazones, see: Lobana *et al.* (2009 ▶). For thio­semicarbazones as ligands in catalysis, see: Xie *et al.* (2010 ▶). For related structures, see: Anderson *et al.* (2012 ▶, 2013*a* ▶,*b* ▶). For standard bond lengths, see: Allen *et al.* (1987 ▶).
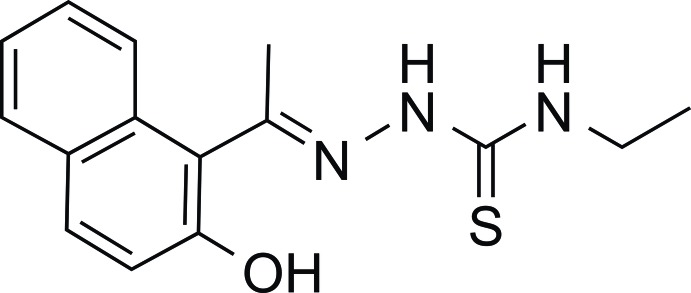


## Experimental   

### 

#### Crystal data  

C_15_H_17_N_3_OS
*M* *_r_* = 287.38
Triclinic, 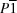

*a* = 8.8988 (7) Å
*b* = 9.2993 (8) Å
*c* = 9.4821 (5) Å
α = 92.525 (6)°
β = 113.034 (7)°
γ = 93.990 (7)°
*V* = 718.18 (10) Å^3^
*Z* = 2
Cu *K*α radiation
μ = 1.99 mm^−1^
*T* = 173 K
0.42 × 0.22 × 0.14 mm

#### Data collection  

Agilent Eos Gemini diffractometer
Absorption correction: multi-scan (*CrysAlis PRO* and *CrysAlis RED*; Agilent, 2012 ▶) *T*_min_ = 0.429, *T*_max_ = 1.000
4327 measured reflections
2710 independent reflections
2365 reflections with *I* > 2σ(*I*)
*R*_int_ = 0.032

#### Refinement  

*R*[*F* ^2^ > 2σ(*F* ^2^)] = 0.054
*wR*(*F* ^2^) = 0.153
*S* = 1.06
2710 reflections
184 parameters
H-atom parameters constrained
Δρ_max_ = 0.41 e Å^−3^
Δρ_min_ = −0.41 e Å^−3^

### 

Data collection: *CrysAlis PRO* (Agilent, 2012 ▶); cell refinement: *CrysAlis PRO*; data reduction: *CrysAlis RED* (Agilent, 2012 ▶); program(s) used to solve structure: *SUPERFLIP* (Palatinus & Chapuis, 2007 ▶; Palatinus & van der Lee, 2008 ▶; Palatinus *et al.*, 2012 ▶).; program(s) used to refine structure: *SHELXL2012* (Sheldrick, 2008 ▶); molecular graphics: *OLEX2* (Dolomanov *et al.*, 2009 ▶); software used to prepare material for publication: *OLEX2*.

## Supplementary Material

Crystal structure: contains datablock(s) I. DOI: 10.1107/S1600536814012057/bt6981sup1.cif

Structure factors: contains datablock(s) I. DOI: 10.1107/S1600536814012057/bt6981Isup2.hkl

Supporting information file. DOI: 10.1107/S1600536814012057/bt6981Isup3.cml

CCDC reference: 1005053

Additional supporting information:  crystallographic information; 3D view; checkCIF report

## Figures and Tables

**Table 1:** Hydrogen-bond geometry (Å, °)

*D*—H⋯*A*	*D*—H	H⋯*A*	*D*⋯*A*	*D*—H⋯*A*
O1—H1⋯S1^i^	0.84	2.40	3.2349 (17)	171
C14—H14*A*⋯O1^ii^	0.99	2.49	3.474 (4)	171
N2—H2⋯S1^iii^	0.88	2.79	3.548 (2)	145

Symmetry codes: (i) 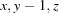; (ii) 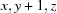; (iii) 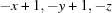.

**Fig. 1.**
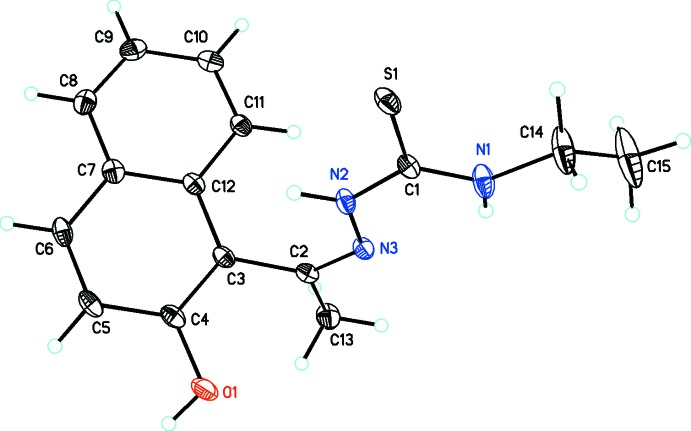
ORTEP drawing of C15H17N3OS, showing the labeling scheme with 30% probability displacement ellipsoids.

**Fig. 2.**
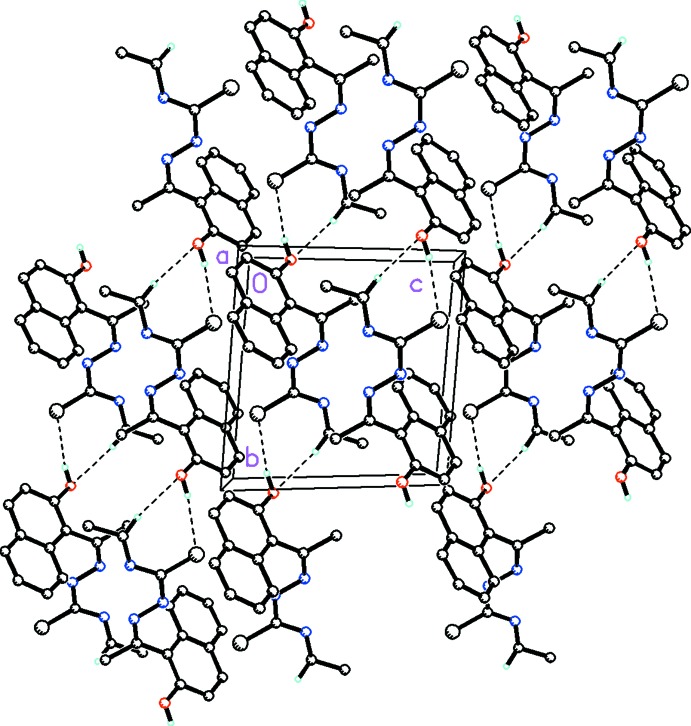
Molecular packing for the title compound viewed along the a axis. Dashed lines indicate weak O1—H1···S1 and C14—H14A···O1 intermolecular interactions forming infinite polymeric chains along [010].

**Fig. 3.**
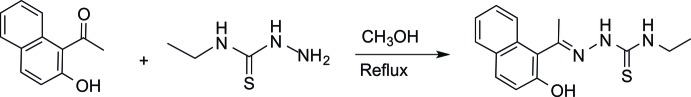
Reaction scheme.

## supplementary crystallographic information

### 1. Comment

Thiosemicarbazones are a versatile class of ligands that have been studied for their biological activity (Chellan *et al.*, 2010), interesting binding motifs (Lobana *et al.*, 2009), and their use as ligands in catalysis (Xie *et al.*, 2010). We have previously reported the structure of three similar novel thiosemicarbazones (Anderson *et al.*, 2012; Anderson *et al.*, 2013*a*; Anderson *et al.*, 2013*b*). Here, we report the synthesis and crystal structure of a new novel thiosemicarbazone ligand starting with 2'-hydroxy-1'-acetonaphthone and 4-ethyl-thio-semicarbazide, C_15_H_17_N_3_OS.

In the title compound, the dihedral angle between the mean planes of the 2-hydroxynapthyl ring and the hydrazinecarbothioamide group (N3/N2/C1/S1/N1) is 73.7 (3)°. Bond lengths are in normal ranges (Allen *et al.*, 1987). In the crystal, weak O1—H1···S1 and C14—H14A···O1 intermolecular interactions and π–π stacking interactions involving one of the hydroxynapthyl rings are observed forming infinite polymeric chains along [010] (Cg2—Cg2 = 3.6648 (14)Å; 2-x, 1-y, -z; Cg2 = C7—C12). In addition, there are N-H···S interactions stabilizing the crystal structure.

### 2. Experimental

A 25 mL round bottom flask was charged with 0.2052 g (1.102 mmol) 2'-hydroxy-1'-acetonaphthone, and 0.1341 g (1.125 mmol) 4-ethyl-thio-semicarbazide and in 5 mL of 1:1 ratio H_2_O to ETOH. The resulting slurry was refluxed for 96 hours (Fig. 3). After reflux the opaque solution was transferred to a separatory funnel to which dichloromethane and water were added. The organic layer was separated and the aqueous layer was extracted twice with 5 mL DCM. The organic layers were combined, washed with brine and dried with magnesium sulfate, and the solvent was removed in vacuo to yield a colorless oil. The oil was the dissolved in minimal 343° K acetonitrile and left to slowly cool to 273° K, after 24 days colorless crystals were observed. m.p. 444–446 K.

### 3. Refinement

All of the H atoms were placed in their calculated positions and then refined using the riding model with Atom—H lengths of 0.95Å (CH), 0.99Å (CH_2_), 0.98Å (CH_3_), 0.88Å (NH) or 0.84Å (OH). Isotropic displacement parameters for these atoms were set to 1.2 (CH, CH_2_, NH) or 1.5 (CH_3_, OH) times *U*_eq_ of the parent atom. Idealised Me refined as rotating group. Idealised tetrahedral OH refined as rotating group.

### Crystal data

**Table:**

C_15_H_17_N_3_OS	*Z* = 2
*M**_r_* = 287.38	*F*(000) = 304
Triclinic, *P*1	*D*_x_ = 1.329 Mg m^−^^3^
*a* = 8.8988 (7) Å	Cu *K*α radiation, λ = 1.54184 Å
*b* = 9.2993 (8) Å	Cell parameters from 1994 reflections
*c* = 9.4821 (5) Å	θ = 4.8–71.3°
α = 92.525 (6)°	µ = 1.99 mm^−^^1^
β = 113.034 (7)°	*T* = 173 K
γ = 93.990 (7)°	Irregular, colourless
*V* = 718.18 (10) Å^3^	0.42 × 0.22 × 0.14 mm

### Data collection

**Table:**

Agilent Eos Gemini diffractometer	2710 independent reflections
Radiation source: Enhance (Cu) X-ray Source	2365 reflections with *I* > 2σ(*I*)
Graphite monochromator	*R*_int_ = 0.032
Detector resolution: 16.0416 pixels mm^-1^	θ_max_ = 71.4°, θ_min_ = 4.8°
ω scans	*h* = −10→10
Absorption correction: multi-scan (*CrysAlis PRO* and *CrysAlis RED*; Agilent, 2012)	*k* = −10→11
*T*_min_ = 0.429, *T*_max_ = 1.000	*l* = −11→9
4327 measured reflections	

### Refinement

**Table:**

Refinement on *F*^2^	Primary atom site location: structure-invariant direct methods
Least-squares matrix: full	Hydrogen site location: inferred from neighbouring sites
*R*[*F*^2^ > 2σ(*F*^2^)] = 0.054	H-atom parameters constrained
*wR*(*F*^2^) = 0.153	*w* = 1/[σ^2^(*F*_o_^2^) + (0.0898*P*)^2^ + 0.2589*P*] where *P* = (*F*_o_^2^ + 2*F*_c_^2^)/3
*S* = 1.06	(Δ/σ)_max_ < 0.001
2710 reflections	Δρ_max_ = 0.41 e Å^−^^3^
184 parameters	Δρ_min_ = −0.41 e Å^−^^3^
0 restraints	

### Special details

**Table:**

Geometry. All esds (except the esd in the dihedral angle between two l.s. planes) are estimated using the full covariance matrix. The cell esds are taken into account individually in the estimation of esds in distances, angles and torsion angles; correlations between esds in cell parameters are only used when they are defined by crystal symmetry. An approximate (isotropic) treatment of cell esds is used for estimating esds involving l.s. planes.

### Fractional atomic coordinates and isotropic or equivalent isotropic displacement parameters (Å^2^)

**Table:**

	*x*	*y*	*z*	*U*_iso_*/*U*_eq_	
S1	0.53047 (7)	0.69555 (5)	0.11487 (6)	0.0286 (2)	
O1	0.6330 (2)	0.03677 (17)	0.2160 (2)	0.0360 (4)	
H1	0.6056	−0.0495	0.1793	0.054*	
N1	0.6310 (3)	0.6503 (2)	0.4127 (2)	0.0342 (5)	
H1A	0.6824	0.5980	0.4893	0.041*	
N2	0.6777 (2)	0.47722 (19)	0.2599 (2)	0.0242 (4)	
H2	0.6733	0.4430	0.1702	0.029*	
N3	0.7469 (2)	0.40209 (19)	0.3892 (2)	0.0234 (4)	
C1	0.6168 (3)	0.6042 (2)	0.2735 (2)	0.0226 (5)	
C2	0.8136 (3)	0.2868 (2)	0.3759 (3)	0.0230 (5)	
C3	0.8262 (3)	0.2283 (2)	0.2325 (2)	0.0219 (4)	
C4	0.7379 (3)	0.1000 (2)	0.1585 (3)	0.0251 (5)	
C5	0.7515 (3)	0.0388 (2)	0.0254 (3)	0.0284 (5)	
H5	0.6860	−0.0475	−0.0267	0.034*	
C6	0.8583 (3)	0.1036 (2)	−0.0279 (3)	0.0278 (5)	
H6	0.8656	0.0625	−0.1182	0.033*	
C7	0.9594 (3)	0.2317 (2)	0.0491 (3)	0.0249 (5)	
C8	1.0824 (3)	0.2928 (3)	0.0032 (3)	0.0294 (5)	
H8	1.0948	0.2499	−0.0839	0.035*	
C9	1.1834 (3)	0.4128 (3)	0.0832 (3)	0.0319 (5)	
H9	1.2667	0.4516	0.0527	0.038*	
C10	1.1634 (3)	0.4782 (2)	0.2102 (3)	0.0298 (5)	
H10	1.2323	0.5623	0.2641	0.036*	
C11	1.0455 (3)	0.4222 (2)	0.2576 (3)	0.0245 (5)	
H11	1.0332	0.4684	0.3433	0.029*	
C12	0.9418 (2)	0.2955 (2)	0.1797 (2)	0.0216 (4)	
C13	0.8917 (3)	0.2093 (3)	0.5182 (3)	0.0344 (6)	
H13A	1.0106	0.2152	0.5473	0.052*	
H13B	0.8472	0.1076	0.4985	0.052*	
H13C	0.8686	0.2543	0.6021	0.052*	
C14	0.5673 (4)	0.7823 (4)	0.4484 (3)	0.0555 (9)	
H14A	0.5943	0.8613	0.3931	0.067*	
H14B	0.4462	0.7659	0.4092	0.067*	
C15	0.6293 (6)	0.8273 (5)	0.6067 (4)	0.0845 (15)	
H15A	0.5734	0.9099	0.6224	0.127*	
H15B	0.7473	0.8558	0.6438	0.127*	
H15C	0.6102	0.7475	0.6636	0.127*	

### Atomic displacement parameters (Å^2^)

**Table:**

	*U*^11^	*U*^22^	*U*^33^	*U*^12^	*U*^13^	*U*^23^
S1	0.0383 (4)	0.0169 (3)	0.0243 (3)	0.0057 (2)	0.0052 (2)	0.0014 (2)
O1	0.0362 (9)	0.0208 (8)	0.0520 (11)	−0.0063 (7)	0.0208 (8)	−0.0064 (7)
N1	0.0440 (12)	0.0314 (11)	0.0233 (10)	0.0204 (9)	0.0066 (9)	0.0003 (8)
N2	0.0329 (10)	0.0193 (9)	0.0190 (9)	0.0090 (7)	0.0078 (8)	0.0005 (7)
N3	0.0277 (9)	0.0196 (9)	0.0206 (9)	0.0026 (7)	0.0071 (7)	0.0008 (7)
C1	0.0210 (10)	0.0188 (10)	0.0236 (11)	0.0022 (8)	0.0044 (8)	−0.0017 (8)
C2	0.0221 (10)	0.0173 (10)	0.0273 (11)	0.0010 (8)	0.0075 (9)	0.0023 (8)
C3	0.0218 (10)	0.0158 (9)	0.0249 (11)	0.0050 (8)	0.0051 (8)	0.0021 (8)
C4	0.0215 (10)	0.0164 (10)	0.0331 (12)	0.0054 (8)	0.0054 (9)	0.0015 (8)
C5	0.0245 (11)	0.0184 (10)	0.0339 (12)	0.0052 (8)	0.0026 (9)	−0.0042 (9)
C6	0.0281 (11)	0.0261 (11)	0.0238 (11)	0.0115 (9)	0.0037 (9)	−0.0042 (9)
C7	0.0248 (11)	0.0235 (10)	0.0240 (11)	0.0100 (9)	0.0057 (9)	0.0048 (8)
C8	0.0339 (12)	0.0310 (12)	0.0253 (11)	0.0120 (10)	0.0119 (10)	0.0072 (9)
C9	0.0314 (12)	0.0323 (12)	0.0357 (13)	0.0065 (10)	0.0156 (10)	0.0124 (10)
C10	0.0274 (11)	0.0242 (11)	0.0339 (12)	0.0005 (9)	0.0078 (10)	0.0052 (9)
C11	0.0264 (11)	0.0194 (10)	0.0247 (11)	0.0040 (8)	0.0067 (9)	0.0019 (8)
C12	0.0205 (10)	0.0176 (10)	0.0222 (10)	0.0062 (8)	0.0029 (8)	0.0039 (8)
C13	0.0469 (14)	0.0273 (12)	0.0293 (13)	0.0120 (11)	0.0135 (11)	0.0078 (10)
C14	0.072 (2)	0.0528 (17)	0.0360 (15)	0.0411 (16)	0.0101 (14)	−0.0068 (13)
C15	0.107 (3)	0.074 (2)	0.051 (2)	0.062 (2)	0.002 (2)	−0.0201 (18)

### Geometric parameters (Å, º)

**Table:**

S1—C1	1.695 (2)	C7—C8	1.420 (3)
O1—H1	0.8400	C7—C12	1.418 (3)
O1—C4	1.366 (3)	C8—H8	0.9500
N1—H1A	0.8800	C8—C9	1.372 (4)
N1—C1	1.324 (3)	C9—H9	0.9500
N1—C14	1.468 (3)	C9—C10	1.404 (4)
N2—H2	0.8800	C10—H10	0.9500
N2—N3	1.384 (3)	C10—C11	1.374 (3)
N2—C1	1.355 (3)	C11—H11	0.9500
N3—C2	1.286 (3)	C11—C12	1.423 (3)
C2—C3	1.491 (3)	C13—H13A	0.9800
C2—C13	1.499 (3)	C13—H13B	0.9800
C3—C4	1.378 (3)	C13—H13C	0.9800
C3—C12	1.428 (3)	C14—H14A	0.9900
C4—C5	1.413 (3)	C14—H14B	0.9900
C5—H5	0.9500	C14—C15	1.413 (4)
C5—C6	1.358 (3)	C15—H15A	0.9800
C6—H6	0.9500	C15—H15B	0.9800
C6—C7	1.422 (3)	C15—H15C	0.9800
			
C4—O1—H1	109.5	C9—C8—H8	119.6
C1—N1—H1A	117.7	C8—C9—H9	120.1
C1—N1—C14	124.6 (2)	C8—C9—C10	119.8 (2)
C14—N1—H1A	117.7	C10—C9—H9	120.1
N3—N2—H2	120.5	C9—C10—H10	119.5
C1—N2—H2	120.5	C11—C10—C9	120.9 (2)
C1—N2—N3	118.96 (18)	C11—C10—H10	119.5
C2—N3—N2	117.91 (18)	C10—C11—H11	119.7
N1—C1—S1	123.94 (17)	C10—C11—C12	120.6 (2)
N1—C1—N2	117.0 (2)	C12—C11—H11	119.7
N2—C1—S1	119.01 (16)	C7—C12—C3	119.48 (19)
N3—C2—C3	125.3 (2)	C7—C12—C11	118.3 (2)
N3—C2—C13	117.1 (2)	C11—C12—C3	122.1 (2)
C3—C2—C13	117.53 (19)	C2—C13—H13A	109.5
C4—C3—C2	119.75 (19)	C2—C13—H13B	109.5
C4—C3—C12	119.3 (2)	C2—C13—H13C	109.5
C12—C3—C2	120.65 (18)	H13A—C13—H13B	109.5
O1—C4—C3	117.4 (2)	H13A—C13—H13C	109.5
O1—C4—C5	121.37 (19)	H13B—C13—H13C	109.5
C3—C4—C5	121.2 (2)	N1—C14—H14A	108.7
C4—C5—H5	120.1	N1—C14—H14B	108.7
C6—C5—C4	119.9 (2)	H14A—C14—H14B	107.6
C6—C5—H5	120.1	C15—C14—N1	114.2 (3)
C5—C6—H6	119.4	C15—C14—H14A	108.7
C5—C6—C7	121.3 (2)	C15—C14—H14B	108.7
C7—C6—H6	119.4	C14—C15—H15A	109.5
C8—C7—C6	121.8 (2)	C14—C15—H15B	109.5
C12—C7—C6	118.7 (2)	C14—C15—H15C	109.5
C12—C7—C8	119.4 (2)	H15A—C15—H15B	109.5
C7—C8—H8	119.6	H15A—C15—H15C	109.5
C9—C8—C7	120.8 (2)	H15B—C15—H15C	109.5
			
O1—C4—C5—C6	−179.3 (2)	C5—C6—C7—C12	−2.9 (3)
N2—N3—C2—C3	1.4 (3)	C6—C7—C8—C9	−177.1 (2)
N2—N3—C2—C13	178.41 (19)	C6—C7—C12—C3	1.3 (3)
N3—N2—C1—S1	−179.44 (15)	C6—C7—C12—C11	178.85 (18)
N3—N2—C1—N1	1.5 (3)	C7—C8—C9—C10	−1.3 (3)
N3—C2—C3—C4	−111.9 (2)	C8—C7—C12—C3	−175.52 (19)
N3—C2—C3—C12	74.7 (3)	C8—C7—C12—C11	2.0 (3)
C1—N1—C14—C15	−165.9 (3)	C8—C9—C10—C11	1.2 (4)
C1—N2—N3—C2	−175.50 (18)	C9—C10—C11—C12	0.6 (3)
C2—C3—C4—O1	4.3 (3)	C10—C11—C12—C3	175.3 (2)
C2—C3—C4—C5	−177.72 (19)	C10—C11—C12—C7	−2.1 (3)
C2—C3—C12—C7	175.56 (18)	C12—C3—C4—O1	177.75 (18)
C2—C3—C12—C11	−1.9 (3)	C12—C3—C4—C5	−4.2 (3)
C3—C4—C5—C6	2.8 (3)	C12—C7—C8—C9	−0.3 (3)
C4—C3—C12—C7	2.1 (3)	C13—C2—C3—C4	71.2 (3)
C4—C3—C12—C11	−175.27 (19)	C13—C2—C3—C12	−102.2 (2)
C4—C5—C6—C7	0.9 (3)	C14—N1—C1—S1	3.2 (4)
C5—C6—C7—C8	173.9 (2)	C14—N1—C1—N2	−177.7 (3)

### Hydrogen-bond geometry (Å, º)

**Table:**

*D*—H···*A*	*D*—H	H···*A*	*D*···*A*	*D*—H···*A*
O1—H1···S1^i^	0.84	2.40	3.2349 (17)	171
C14—H14*A*···O1^ii^	0.99	2.49	3.474 (4)	171
N2—H2···S1^iii^	0.88	2.79	3.548 (2)	145

Symmetry codes: (i) *x*, *y*−1, *z*; (ii) *x*, *y*+1, *z*; (iii) −*x*+1, −*y*+1, −*z*.
